# Childhood trauma and PMOS-related quality of life: perceived stress statistically accounts for part of the association

**DOI:** 10.3389/fpsyg.2026.1846360

**Published:** 2026-10-28

**Authors:** Marleen M. M. Santantonio, Marek Preiss, David Levcik

**Affiliations:** School of Psychology, University of New York in Prague (UNYP), Prague, Czechia

**Keywords:** childhood trauma, indirect association, mental health, perceived stress, polyendocrine metabolic ovarian syndrome, quality of life

## Abstract

**Introduction:**

Polyendocrine metabolic ovarian syndrome (PMOS), formerly known as polycystic ovary syndrome (PCOS), is among the most prevalent endocrine disorders, and it is associated with substantial impairment in quality of life. Emerging evidence suggests that childhood trauma and stress-related processes may contribute to psychological burden in women with PMOS, but these links are poorly understood. The present study examines the relationship between childhood trauma, perceived stress, and PMOS-related quality of life.

**Methods:**

In a cross-sectional online survey, 206 adult women reporting a prior PMOS diagnosis completed three validated questionnaires: the Polycystic Ovary Syndrome Quality of Life Questionnaire (PCOSQ), the Perceived Stress Scale (PSS), and the Childhood Trauma Questionnaire-Short Form (CTQ-SF).

**Results:**

The results indicate significant correlations: higher levels of childhood trauma are associated with poorer PMOS-related quality of life (*ρ* = −0.38, *ρ* < 0.001) and increased perceived stress (*ρ* = 0.40, *ρ* < 0.001). Regression analyses showed that higher perceived stress was significantly associated with lower PCOSQ scores (*β*s = −0.54, p < 0.001). Additionally, an indirect-association analysis showed that perceived stress statistically accounted for part of the association between childhood trauma and PMOS-related quality of life (*β* = −0.18, ρ < 0.001).

**Discussion:**

Women with greater trauma histories reported higher perceived stress, and higher perceived stress was associated with poorer quality of life. These findings support further prospective and intervention research examining whether assessment of trauma history and perceived stress may help identify women at greater risk of poorer PMOS-related quality of life and whether trauma-informed approaches can improve psychosocial outcomes.

## 1 Introduction

Polyendocrine metabolic ovarian syndrome (PMOS), formerly known as Polycystic ovary syndrome (PCOS), is the current consensus name for the condition following the 2026 global nomenclature change (Teede et al., 2026). The syndrome affects approximately 8–13% of women of reproductive age worldwide (Hoeger et al., 2021), while a recent global meta-analysis estimated an overall prevalence of 9.2%, with estimates varying according to diagnostic criteria (Salari et al., 2024). Beyond its reproductive manifestations, PMOS is associated with significant metabolic and psychological burden, with long-term implications for women’s physical health, emotional well-being, and quality of life (Joham et al., 2022). Accordingly, PMOS is increasingly approached from a biopsychosocial perspective, reflecting the coexistence of endocrine dysregulation and psychological distress (Moran et al., 2010).

Women with PMOS consistently report substantial psychological burden, including elevated anxiety and depressive symptoms compared with women without the condition (Podfigurna-Stopa et al., 2015; Alur-Gupta et al., 2019), while higher perceived stress has also been observed in PMOS samples relative to controls (Marschalek et al., 2023). Anxiety symptoms were reported in 76.5% and depressive symptoms in 28% of women with PMOS in one clinical sample (Alur-Gupta et al., 2019). Together, these findings underscore the psychological burden associated with PMOS and its relationship with poorer quality of life, while also pointing to stress-related processes as a potentially salient feature of the condition. Physiological studies also suggest altered stress regulation in PMOS (Benson et al., 2009; Vojnović Milutinović et al., 2011), although evidence regarding hypothalamic–pituitary–adrenal (HPA) axis alterations is mixed and may partly reflect factors such as obesity (Pasquali and Gambineri, 2012). Within this broader biopsychosocial context, population-based evidence indicates that adverse childhood experiences (ACEs) are more frequently reported by women with self-reported PMOS compared to women without PMOS, including psychological, physical, and sexual abuse (Tay et al., 2020). A separate study of psychiatrically healthy women found that emotional abuse remained associated with probable PMOS once other forms of childhood maltreatment were taken into account (Pringle et al., 2022).

While stress-related dysregulation has been documented in PMOS, considerably less attention has been given to factors that may shape individual differences in stress perception among women with the condition. More broadly, evidence from non-PMOS trauma-related populations suggests that trauma exposure and trauma-related psychopathology may be associated with differences in HPA-axis regulation, although findings vary across studies (Kuhlman et al., 2015; Speer et al., 2019). The potential relevance of trauma-related stress dysregulation to PMOS remains an important area of investigation. Recent cross-sectional evidence further indicates that severe ACE exposure (≥4 ACEs) was associated with approximately twofold higher adjusted odds of PMOS in young adulthood (Sharma et al., 2025).

Beyond condition-specific outcomes, evidence from an adult clinical population also links childhood maltreatment with poorer quality of life. In treatment-seeking adults with generalized social anxiety disorder, greater childhood maltreatment was associated with poorer quality of life and functioning, while exposure to multiple maltreatment types showed an additive association with these outcomes (Simon et al., 2009). Developmental research further suggests that early-life stress may have lasting implications for affective and stress-related functioning, including emotion regulation, and emotional and social-information processing (Gunnar, 2020; Gee, 2021; Smith and Pollak, 2020). Importantly, responses to early adversity are heterogeneous: similar exposures may be associated with different outcomes, while different forms of adversity may also converge on similar outcomes (Gunnar, 2020; Gee, 2021). This heterogeneity supports considering childhood trauma as a contextual factor that may contribute to individual differences in perceived stress and quality of life, without implying a direct causal pathway.

Despite this theoretical convergence, the role of childhood trauma in relation to perceived stress and quality of life among women with PMOS remains underexplored. The psychological literature on PMOS has largely focused on quality of life, depression, and anxiety (Yin et al., 2021), whereas, comparatively, research addressing childhood adversity remains limited (Berndt et al., 2025). In a recent systematic review, only two studies were found examining childhood adversity in relation to PMOS, and the resulting body of evidence was insufficient to draw firm conclusions about this association (Berndt et al., 2025). Within this narrow literature, ACEs and perceived stress have mainly been examined in relation to psychiatric morbidity (Tay et al., 2020), while the latest findings have linked childhood trauma to poorer PMOS-related quality of life, with coping explored as a potential explanatory mechanism (Schneider and Chin, 2025). Collectively, these observed patterns underscore that the relationships among quality of life in women with PMOS, perceived stress, and childhood trauma remain relatively less well-characterized. Given that PMOS-related quality of life and perceived stress may also vary according to sociodemographic and illness-related characteristics, these factors were considered alongside childhood trauma as potential covariates. Specifically, age, ethnicity, marital status, occupation, educational level, and duration of PMOS symptoms were included, as previous research has linked demographic and clinical characteristics with quality-of-life and psychosocial outcomes in women with PMOS (Rzońca et al., 2018; Damone et al., 2019; Sendur and Yildiz, 2021; Tabassum et al., 2021; Kaur et al., 2023).

The present study investigated associations among PMOS-related quality of life, perceived stress, and childhood trauma within a cross-sectional design. We hypothesized that (1) greater exposure to childhood trauma would be associated with higher levels of perceived stress; (2) greater exposure to childhood trauma would be associated with poorer PMOS-related quality of life; and (3) perceived stress would statistically account for part of the association between childhood trauma and PMOS-related quality of life.

## 2 Methods

### 2.1 Participants

Participants (*N* = 206) were recruited through social media platforms and online support groups dedicated to PMOS. Eligibility criteria included: (1) a self-reported prior diagnosis of PMOS by a healthcare professional, (2) age between 18 and 45 years, (3) absence of diagnosed severe mental health conditions, such as major depression, bipolar disorder, or schizophrenia, or significant physiological comorbidities commonly associated with PMOS, such as type 2 diabetes or cardiovascular disease, and (4) sufficient English proficiency to comprehend study materials and independently complete the questionnaires.

Participants were asked to report whether they had previously received a PMOS diagnosis from a healthcare professional; no medical records were obtained to independently verify the diagnosis. The exclusion of severe psychiatric conditions and major physiological comorbidities was intended to reduce potential confounding from conditions that could independently affect perceived stress and quality of life; however, these eligibility criteria could not eliminate residual clinical confounding. An upper age limit of 45 years was imposed to reduce potential confounding effects of perimenopausal and menopausal hormonal changes, which may influence both PMOS symptom expression and stress-related psychological responses. Participants were also required to confirm their proficiency in English to ensure they could complete the questionnaire independently. English language proficiency was assessed through a self-reported screening question in the eligibility section: “Do you have sufficient proficiency in English to understand the study materials and complete the questionnaires independently?” Only those who confirmed this criterion proceeded to the main questionnaire. This requirement was included to ensure comprehension accuracy, as all validated scales—The Polycystic Ovary Syndrome Quality of Life Questionnaire (PCOSQ), Perceived Stress Scale (PSS), and Childhood Trauma Questionnaire-Short Form—were administered in English. Ensuring adequate English proficiency minimized potential misinterpretation of questionnaire items and maintained the validity of self-reported data. Of the initial 252 submissions, 35 were excluded at the eligibility stage through automated branching logic: three respondents did not confirm a prior PMOS diagnosis by a healthcare professional, one did not meet the 18–45-year age criterion, and 31 reported an exclusionary severe mental-health condition or a significant physiological comorbidity. Eleven additional submissions were excluded during post-eligibility data-quality screening. Ten of these participants had initiated the questionnaire but left sufficient items unanswered for missingness to exceed the defined measure-specific limits: more than 20% missing within the relevant PCOSQ or CTQ-SF domain/subscale, or more than one missing item on the 10-item PSS. One further submission was excluded as an exact duplicate response. This resulted in a final analytic sample of 206 participants.

### 2.2 Procedure

Data collection involved five stages: recruitment, obtaining informed consent, completing the questionnaire, implementing data protection measures, and data screening. To increase accessibility, the study was described concisely in posts shared on social media, in online support groups for PMOS, and through health networks. Each post included a link to the online questionnaire, and posts were published regularly to achieve an adequate sample size. Informed consent documents outlined the study’s objectives, methods, risks, and confidentiality measures. Only participants who provided informed consent were included. No financial compensation, reimbursement, course credit, or other incentive was provided for participation. In alignment with the study’s online recruitment strategy, Google Forms was used to expedite the questionnaire distribution, enabling participants’ remote and independent completion. The questionnaire typically required 15 to 20 min to complete. Google Forms was configured not to collect participants’ email addresses, and respondents were not required to sign in to a Google account to complete the survey. No direct identifying information was requested. The response file contained an automatically generated submission timestamp but no account-related identifiers. The linked response spreadsheet was stored in Google Drive with restricted access and was accessible only to the principal investigators. Participants were informed of the confidentiality protocol before providing consent and, given the sensitive nature of the childhood-trauma questions, they were exempt from addressing any prompt deemed discomfort-inducing and allowed to discontinue participation at any time. The informed-consent content further suggested reaching out to a trusted individual or professional for assistance if the completion process elicited emotions or memories warranting additional support. Specific psychological-support or crisis-service contact details were not provided. During data analysis, responses were checked for completeness and duplicate submissions. Cases exceeding the measure-specific missingness limits described above were excluded rather than imputed, and one exact duplicate submission was removed. Participants with only minimal item-level missingness were retained, and missing values were imputed using the mean of that participant’s completed items within the relevant domain or subscale. For the PCOSQ and CTQ-SF subscales, imputation was limited to cases with 20% or fewer missing items within the relevant domain or subscale. For the 10-item PSS, no more than one missing item was imputed. Item-level imputation was required for 10 participants (4.9% of the final analytic sample), with one item imputed per participant: three cases involved the PCOSQ, three the PSS, and four the CTQ-SF. Prior to data collection, ethical approval for the study (ref. nr. 2025-0108) was obtained from the University of New York in Prague in accordance with Czech and EU guidelines for research involving human participants. All procedures performed in this study involving human participants were in accordance with the ethical standards of the institutional and/or national research committee and with the 1964 Declaration of Helsinki and its later amendments, or comparable ethical standards.

### 2.3 Measures

Sociodemographic and illness-related information collected included age, ethnicity, marital status, occupation, educational level, and duration of PMOS symptoms. Detailed clinical variables such as BMI, PMOS phenotype or symptom severity, current medication or treatment status, and dimensional psychiatric symptom measures were not collected.

The study examines three primary variables: PMOS-related quality of life, perceived stress over the past month, and childhood trauma. Each variable was assessed using a validated self-assessment instrument demonstrating strong internal consistency.

The PCOSQ is a validated self-report instrument developed by Cronin et al. (1998) to assess health-related quality of life in women with PMOS. It consists of 26 items organized into five domains: emotional well-being, hirsutism (excessive hair growth in androgen-sensitive areas), concerns about excess weight, infertility, and menstrual irregularities. Although the PCOSQ captures five distinct symptom-related domains, the present study used the overall PCOSQ score as the primary outcome because the research question concerned global PMOS-related quality of life rather than domain-specific symptom burden. Previous research has similarly calculated an overall PCOSQ score across the five domains (McCook et al., 2005), while subsequent validation work has reported high internal consistency, test–retest reliability, and evidence of validity for the total score (Böttcher et al., 2018). An example item is “Feel a lack of control over the situation with PCOS?”. Items are rated on a seven-point Likert scale using two response formats. Discomfort caused by symptoms is rated from “severe problem” (1) to “no problem” (7), while symptom frequency is rated from “all of the time” (1) to “none of the time” (7). The maximum total score is 182, with domain scores calculated as the mean of item responses within each domain. Higher scores reflect better PMOS-related quality of life. In a United Kingdom validation study by Jones (2004), all five domains demonstrated strong internal consistency (Cronbach’s *α* = 0.70–0.97) and excellent test–retest reliability (intra-class correlation coefficients = 0.89–0.95). In the present sample, the PCOSQ demonstrated excellent internal consistency, with a Cronbach’s alpha of 0.91. The PCOSQ is widely used to evaluate the impact of PMOS on quality of life and to measure treatment outcomes.

The PSS is a validated self-report instrument developed by Cohen et al. (1983); the present study used the widely adopted 10-item version described by Cohen and Williamson (1988), which assesses perceived stress during the previous month. An example item is “In the last month, how often have you felt nervous and stressed?”. Each item is rated on a five-point Likert scale, ranging from 0 (Never) to 4 (Very often). Scores are calculated by summing item responses, with specific items reverse-coded prior to summation. The total score ranges from 0 to 40, with higher scores indicating greater perceived stress. The 10-item version of the PSS demonstrated adequate internal consistency in its preliminary evaluation (Cronbach’s alpha = 0.78; Cohen and Williamson, 1988). In the present sample, the PSS demonstrated good internal consistency, with a Cronbach’s alpha of 0.81. Although the PSS does not provide diagnostic information, it yields valuable insights into subjective stress perceptions and underscores the variability in individual responses to external events.

The CTQ-SF is a validated self-report instrument developed by Bernstein et al., 2003. It consists of 28 items designed to assess exposure to child neglect and abuse across five domains: emotional abuse, physical abuse, sexual abuse, emotional neglect, and physical neglect. An example item is “I’ve been hit so hard that it left bruises and marks”. Each item is rated on a five-point Likert scale from 1 (“Never true”) to 5 (“Very often true”). Domain scores are calculated by summing responses, with certain items requiring reverse coding. Each domain score ranges from 5 to 25. Higher Likert-scale scores indicate greater reported severity of abuse or neglect. Cut-off scores categorize childhood trauma severity into four levels: None (Minimal), Low (to Moderate), Moderate (to Severe), and Severe (to Extreme). These thresholds dichotomize participants into positive (moderate–severe) and negative (none-mild) exposure groups. The CTQ-SF also includes a Minimization/Denial Scale to identify underreporting of maltreatment, scored from 0 to 3 based on specific items endorsed as “Very often true.” The five-domain structure of the CTQ-SF enables sub-analyses to assess the differential contribution of trauma subtypes to distress in women with PMOS. This approach allows for the analysis of each trauma subtype and its association with perceived stress levels and PMOS-related quality of life. In a meta-analysis of reliability estimates across 39 independent samples, Badenes-Ribera et al. (2024) found that the CTQ-SF demonstrated generally good reliability across studies. The average Cronbach’s alpha for the total score was 0.891 (95% CI: 0.868–0.910), with subscale values ranging from 0.656 (Physical Neglect) to 0.916 (Sexual Abuse). McDonald’s Omega had a total score of 0.800 (95% CI: 0.800–0.800), with subscale values ranging from 0.740 to 0.900. Test–retest reliability for the total score averaged 0.788 (95% CI: 0.635–0.872), with subscale values from 0.668 to 0.709. These findings support the CTQ-SF as a reliable measure of childhood maltreatment. In the present sample, the overall CTQ-SF demonstrated good internal consistency, with a Cronbach’s alpha of 0.90. Internal consistency for the individual subscales was *α* = 0.78 for Physical Abuse, *α* = 0.81 for Emotional Abuse, *α* = 0.92 for Sexual Abuse, *α* = 0.69 for Physical Neglect, and *α* = 0.90 for Emotional Neglect. Due to this scale’s capacity to assess early adversity, the CTQ-SF is widely used in research and clinical settings. The scale’s multidomain structure supports examination of associations between different forms of childhood maltreatment, perceived stress, and PMOS-related quality of life.

### 2.4 Data analysis

Data analysis in this study utilized Jamovi (*Version 2.3.28*) to investigate the relationships among PMOS-related quality of life, perceived stress, and childhood trauma. The analyses included descriptive statistics, correlation analysis, regression models, and indirect-association analysis.

Prior to data analysis, the primary scales administered (PCOSQ, PSS, CTQ-SF) were subjected to the necessary rule-based transformations. For the CTQ-SF, the minimization score was also calculated. In the original scoring, a small subset of participants (8.3%) scored positively on the Minimization/Denial scale, suggesting possible under-reporting of childhood trauma. To evaluate the potential influence of minimization or under-reporting, a conservative sensitivity analysis was conducted by re-screening the archived participant-level Minimization/Denial items and repeating the principal correlation, regression, and indirect-effect analyses after excluding all cases flagged during this re-screening.

Descriptive statistics were calculated to assess central tendency and variability for each scale (PCOSQ, PSS, and CTQ-SF), including mean, median, standard deviation, minimum, and maximum values. Distributional characteristics of PCOSQ, PSS, and CTQ-SF were assessed using the Shapiro–Wilk test, which indicated departures from normality for all three variables, with CTQ-SF exhibiting the greatest departure. Spearman’s rank-order correlation was employed to estimate monotonic associations among PMOS-related quality of life, perceived stress, and childhood trauma. Correlation effect sizes were interpreted using Cohen (1988) guidelines (small *ρ* ≈ 0.10; medium *ρ* ≈ 0.30; large *ρ* ≈ 50). Scatterplots were inspected to assess the form of the associations among the study variables. Secondary exploratory analyses examined the associations of the five CTQ-SF trauma subtypes with perceived stress and PMOS-related quality of life. To account for multiple testing across the 10 subtype–outcome correlations, Holm’s sequential correction was applied across this family of tests (Holm, 1979).

The suitability of the regression models was evaluated using model diagnostics rather than inferred from the marginal distributions of the raw study variables. Residual-versus-fitted plots indicated no major violations of linearity or homoscedasticity. An initial regression model was conducted to examine the association between childhood trauma and perceived stress. Subsequent regression models assessed the independent associations of childhood trauma and perceived stress with PMOS-related quality of life. Age, ethnicity, marital status, occupation, educational level, and duration of PMOS symptoms were included as control variables in each regression model to estimate the associations of perceived stress and childhood trauma with PCOS-related quality of life while accounting for these measured demographic and illness-related characteristics. Collinearity was assessed after running the regression model using the Variance Inflation Factor (VIF) to assess whether multicollinearity was problematic. An indirect-association analysis was conducted to test whether perceived stress statistically accounted for part of the association between childhood trauma and PMOS-related quality of life. The statistical model was theoretically motivated by the relationships among PMOS-related quality of life, perceived stress, and childhood trauma outlined in the Introduction. The primary indirect-association model was unadjusted. To examine whether the indirect statistical association remained after accounting for the same covariates included in the regression analyses, an additional model was estimated including age, ethnicity, marital status, occupation, educational level, and duration of PMOS symptoms as covariates in both the mediator and outcome equations. Nonparametric bootstrapping procedures with 5,000 resamples were implemented. Confidence intervals for indirect statistical associations were calculated using both percentile and bias-corrected bootstrapping. Statistical significance of the indirect association was inferred when the 95% bootstrap confidence interval did not include zero. Model parameters, including unstandardized estimates for indirect, direct, and total association components, as well as standardized coefficients, standard errors, z-values, and bootstrap intervals calculated using both percentile and bias-corrected methods, are reported. All corresponding *p*-values were two-tailed, with *α* = 0.05 set as the significance threshold for *p*-value-based tests. Given the cross-sectional design, these analyses are interpreted as patterns of association rather than evidence of causal pathways.

## 3 Results

### 3.1 Descriptive statistics

The study sample consisted of 206 participants with diverse demographics and illness-related characteristics, as presented in Table 1. Most participants were aged 18–30 years (68.9%), were single (56.8%), and identified as Caucasian/White (49.5%). Full-time employment (39.3%) and student status (35.4%) were the most common occupational categories, and 47.6% of participants reported PMOS symptoms lasting more than 5 years. The sample consisted of self-selected participants recruited online, primarily from PMOS support groups and social media.

**Table 1:** Demographic variables.

Variable	Category	Count	% of total
Age group	18–30 years	142	68.9%
	31–45 years	64	31.1%
Ethnicity	Caucasian/White	102	49.5%
	Asian	28	13.6%
	Hispanic or Latino/a	24	11.7%
	Black or African American	22	10.7%
	Multiracial/Others	30	14.6%
Marital status	Single	117	56.8%
	Married/Common	79	38.3%
	Separated/Divorced	10	4.9%
Occupation	Employed full-time	81	39.3%
	Student	73	35.4%
	Employed part-time	24	11.7%
	Homemaker/Unemployed	28	13.6%
Education	Completed College /University Degree	115	55.8%
	Some College/Some University	66	32.0%
	Completed High School	23	11.2%
	Primary school or less	2	1.0%
Duration of PMOS symptoms	More than 5 years	98	47.6%
	1–5 year(s)	61	29.6%
	Less than 1 year	47	22.8%

To assess the range and distribution of key study variables, we examined participants’ PMOS-related quality of life, childhood trauma exposure, and perceived stress, with descriptive statistics presented in Table 2. PCOSQ scores ranged from 32 to 154, with a mean of 81 (SD = 26.20) and a median of 81, reflecting substantial variability in quality of life. PSS scores ranged from 8 to 37, with a mean of 23 (SD = 6.17) and a median of 24, indicating limited variability in perceived stress. CTQ-SF scores ranged from 25 to 104, with a mean of 52.3 (SD = 15.40) and a median of 50, demonstrating moderate variability in childhood trauma exposure. The Shapiro–Wilk test indicated significant departures from normality for all variables (PCOSQ: *W* = 0.980, *p* = 0.005; PSS: *W* = 0.985, *p* = 0.025; CTQ-SF: *W* = 0.966, *p* < 0.001).

**Table 2:** Descriptive statistics for CTQ-SF, PSS, and PCOSQ.

Variable	*M*	Mdn	SD	Min	Max	Shapiro–Wilk *W*	Shapiro–Wilk *p*
PCOSQ	81	81	26.20	32	154	0.980	0.005
PSS	23	24	6.17	8	37	0.985	0.025
CTQ-SF	52.3	50	15.40	25	104	0.966	<0.001

PCOSQ, Polycystic Ovary Syndrome Health-Related Quality of Life Questionnaire; PSS, Perceived Stress Scale; CTQ-SF, Childhood Trauma Questionnaire–Short Form; *M*, mean; Mdn, median; SD, standard deviation; *W*, Shapiro–Wilk statistic.

### 3.2 Correlational analyses

We next examined the associations among PMOS-related quality of life, perceived stress, and childhood trauma to determine how these variables related to one another, with Spearman’s correlations presented in Table 3. Results indicated that poorer PMOS-related quality of life (PCOSQ) was correlated with higher perceived stress (PSS) and greater childhood trauma (CTQ-SF). A medium negative correlation was found between PCOSQ and CTQ-SF (*ρ* = −0.376, *p* < 0.001), indicating that higher levels of childhood trauma are associated with poorer PMOS-related quality of life. A medium positive correlation was observed between PSS and CTQ-SF (*ρ* = 0.399, *p* < 0.001), suggesting that greater childhood trauma corresponds to higher perceived stress. The largest association was observed between PCOSQ and PSS (*ρ* = −0.522, *p* < 0.001), indicating that higher perceived stress is associated with poorer PMOS-related quality of life.

**Table 3:** Spearman’s correlation matrix for CTQ-SF, PSS, and PCOSQ.

Variable	PCOSQ (*ρ*)	PSS (*ρ*)
PSS	−0.522***	
CTQ-SF	−0.376***	0.399***

PCOSQ, Polycystic Ovary Syndrome Health-Related Quality of Life Questionnaire; PSS, Perceived Stress Scale; CTQ-SF, Childhood Trauma Questionnaire–Short Form; *ρ*, Spearman’s rank correlation coefficient; *p < 0.001.

The relationships among PMOS-related quality of life, perceived stress, and childhood trauma were then explored to visualize how these variables covary, with the correlation matrix scatterplot illustrated in Figure 1. The top-left plot (PCOSQ vs. PSS) demonstrates a clear negative linear relationship, indicating that higher perceived stress is significantly associated with lower PCOSQ scores. The bottom-left plot (PCOSQ vs. CTQ-SF) shows a weaker negative trend, suggesting that greater childhood trauma is modestly associated with reduced PMOS-related quality of life. The bottom-right plot (PSS vs. CTQ-SF) displays a positive linear relationship, indicating that higher childhood trauma is associated with greater perceived stress.

**Figure 1:**
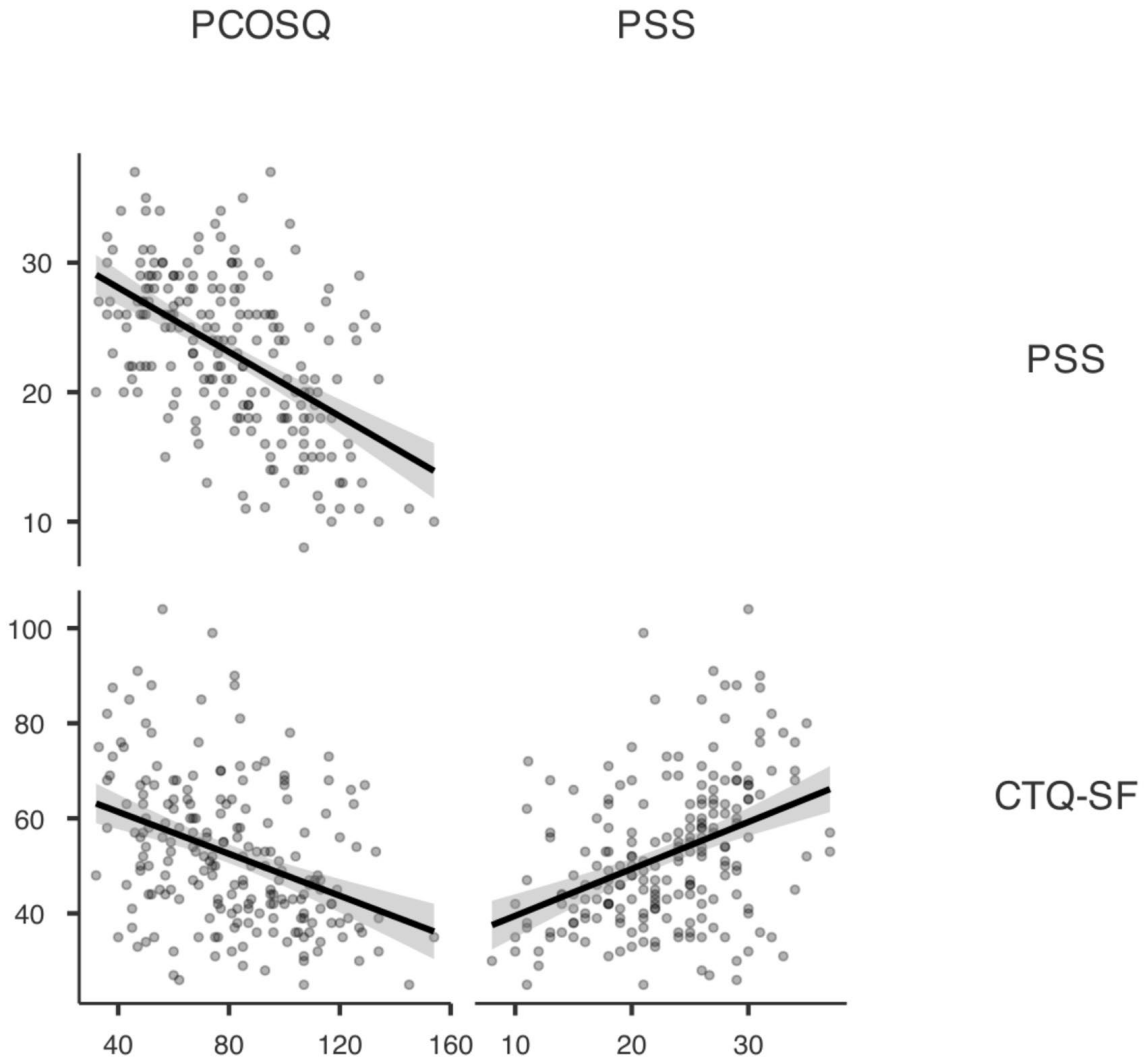
Childhood trauma is positively related to perceived stress and negatively related to PMOS-related quality of life. Spearman’s rank-order correlations (*ρ*; two-tailed *p*-values) are shown for bivariate, nonparametric associations between childhood trauma (CTQ-SF), perceived stress (PSS), and PMOS-related quality of life (PCOSQ) in a sample of 206 participants. Raw total scores are plotted on the *x*- and *y*-axes, with fitted trend lines illustrating the direction and strength of each association (CTQ-SF, Childhood Trauma Questionnaire-Short Form; PSS, Perceived Stress Scale; PCOSQ, Polycystic Ovary Syndrome Quality of Life Questionnaire).

Secondary exploratory analyses examined the associations of specific childhood trauma subtypes with PMOS-related quality of life and perceived stress, with the correlations for each subtype presented in Table 4. Holm’s sequential correction across the 10 subtype–outcome correlations did not alter the pattern of statistical significance. Emotional abuse showed the largest observed negative correlation with PCOSQ (*ρ* = −0.362, *p* < 0.001), followed by emotional neglect (*ρ* = −0.356, *p* < 0.001), physical neglect (*ρ* = −0.233, *p* < 0.001), and physical abuse (*ρ* = −0.230, *p* < 0.001). For PSS, emotional abuse showed the largest observed positive correlation (*ρ* = 0.416, *p* < 0.001), followed by emotional neglect (*ρ* = 0.331, *p* < 0.001), physical neglect (*ρ* = 0.255, *p* < 0.001), and physical abuse (*ρ* = 0.168, *p* = 0.016). No statistically significant association was detected between sexual abuse and either PCOSQ (*ρ* = −0.051, *p* = 0.469) or PSS (*ρ* = 0.021, *p* = 0.769).

**Table 4:** Secondary exploratory correlations between CTQ-SF subtypes, PCOSQ, and PSS.

CTQ-SF subscales	PCOSQ (*ρ*)	PSS (*ρ*)
Physical abuse	−0.230 (*p* < 0.001)	0.168 (*p* = 0.016)
Emotional abuse	−0.362 (*p* < 0.001)	0.416 (*p* < 0.001)
Sexual abuse	−0.051 (*p* = 0.469)	0.021 (*p* = 0.769)
Physical neglect	−0.233 (*p* < 0.001)	0.255 (*p* < 0.001)
Emotional neglect	−0.356 (*p* < 0.001)	0.331 (*p* < 0.001)

Spearman’s *ρ* is reported. Nominal *p*-values are shown. Holm’s sequential correction was applied across the 10 subtype–outcome correlations; the pattern of statistical significance was unchanged. PCOSQ, Polycystic Ovary Syndrome Health-Related Quality of Life Questionnaire; PSS, Perceived Stress Scale; CTQ-SF, Childhood Trauma Questionnaire–Short Form; *ρ*, Spearman’s rank correlation coefficient.

### 3.3 Regression models

To assess whether the associations identified in the correlation analyses were also observed in regression models, we examined whether childhood trauma was associated with perceived stress and whether childhood trauma and perceived stress were independently associated with PMOS-related quality of life, with results provided in Table 5. The first model demonstrated a significant positive association between CTQ-SF and PSS, accounting for 33.2% of the variance (*R*^2^ = 0.332). Childhood trauma was significantly positively associated with perceived stress (*β* = 0.374, 95% CI [0.233, 0.516], *p* < 0.001), indicating that higher levels of childhood trauma were associated with higher perceived stress among women with PMOS. The second model revealed a significant negative association between CTQ-SF and PCOSQ, explaining 30.7% of the variance (*R*^2^ = 0.307). In this model, greater childhood trauma was significantly associated with lower PCOSQ scores (*β* = −0.391, 95% CI [−0.536, −0.247], *p* < 0.001). The third model demonstrated a significant negative association between PSS and PCOSQ, accounting for 41.4% of the variance (*R*^2^ = 0.414). Higher perceived stress was significantly associated with lower PMOS-related quality of life (*β* = −0.538, 95% CI [−0.671, −0.406], *p* < 0.001). These findings indicate that the associations among childhood trauma, perceived stress, and PMOS-related quality of life remained significant in the regression models.

**Table 5:** Linear regression model fit measures for PSS and PCOSQ.

Model	*R* ^2^	*βₛ*	95% CIs (L, U)	*p*
PSS model: CTQ-SF	0.332	0.374	0.233, 0.516	<0.001
PCOSQ model: CTQ-SF	0.307	−0.391	−0.536, −0.247	<0.001
PCOSQ model: PSS	0.414	−0.538	−0.671, −0.406	<0.001

PSS, Perceived Stress Scale; PCOSQ, Polycystic Ovary Syndrome Health-Related Quality of Life Questionnaire; CTQ-SF, Childhood Trauma Questionnaire–Short Form; *R*^2^, coefficient of determination; *βₛ*, standardized regression coefficient; CI, confidence interval; L, lower bound; U, upper bound.

We further evaluated the associations of demographic factors with PMOS-related quality of life and perceived stress while accounting for childhood trauma, with significant demographic associations presented in Table 6. In the first model, PSS was significantly associated with childhood trauma (CTQ-SF) while controlling for demographics. CTQ-SF remained significantly associated with PSS (*β* = 0.374, *p* < 0.001), indicating that higher levels of childhood trauma were associated with increased perceived stress. Age was also a significant factor; participants aged 25–30 (*β* = −0.439, *p* = 0.028), 31–35 (*β* = −0.781, *p* = 0.005), and 36–40 (*β* = −0.739, *p* = 0.019) reported lower stress compared to those aged 18–24. Other demographic variables, including ethnicity, marital status, occupation, education, and duration of PMOS symptoms, were not significantly associated with PSS. In the second model, PCOSQ was significantly associated with CTQ-SF while controlling for demographics. CTQ-SF remained significantly associated with PCOSQ (*β* = −0.391, *p* < 0.001), demonstrating that greater childhood trauma was associated with poorer PMOS-related quality of life. Ethnicity was also significantly associated with PCOSQ scores; participants identifying as Black/African American (*β* = 0.566, *p* = 0.043), Caucasian/White (*β* = 0.547, *p* = 0.009), and Middle Eastern/North African (*β* = 0.852, *p* = 0.033) reported higher PCOSQ scores than those identifying as Asian. No other demographic variables reached significance. In the third model, PCOSQ was significantly associated with PSS while controlling for demographics. Perceived stress remained significantly associated with PCOSQ (*β* = −0.538, *p* < 0.001), indicating that higher stress was strongly associated with poorer quality of life. When PSS was included in the model, none of the demographic variables were significantly associated with PCOSQ. These findings further demonstrate the cross-sectional associations among childhood trauma, perceived stress, and PMOS-related quality of life. Multicollinearity was assessed using VIF and tolerance values, which are shown in Table 7, and indicated no evidence of problematic multicollinearity. Across the three regression models, VIF values ranged from 1.05 to 1.23, remaining well below the commonly accepted thresholds of 5 or 10, indicating no concerns regarding multicollinearity. Similarly, tolerance values ranged from 0.813 to 0.950, exceeding the critical value of 0.2 and likewise indicating no evidence of problematic multicollinearity. VIF values for demographic variables such as age, ethnicity, marital status, occupation, education, and duration of PMOS symptoms were consistently low, suggesting minimal multicollinearity among these factors and with the primary predictors (CTQ-SF and PSS). A similar pattern was observed for CTQ-SF and PSS, indicating no evidence of problematic collinearity between the main variables and demographic controls.

**Table 6:** Regression coefficients for main study variables and significant demographic covariates.

Variable	Model 1: PSS (DV)	Model 2: PCOSQ (DV)	Model 3: PCOSQ (DV)
Main study variables			
CTQ-SF	*β* = 0.374, 95% CI [0.233, 0.516], *p* < 0.001	*β* = −0.391, 95% CI [−0.536, −0.247], *p* < 0.001	—
PSS	—	—	*β* = −0.538, 95% CI [−0.671, −0.406], *p* < 0.001
Demographics			
Age 25–30 (vs. 18–24)	*β* = −0.439, 95% CI [−0.829, −0.049], *p* = 0.028	ns	ns
Age 31–35 (vs. 18–24)	*β* = −0.781, 95% CI [−1.322, −0.240], *p* = 0.005	ns	ns
Age 36–40 (vs. 18–24)	*β* = −0.739, 95% CI [−1.355, −0.122], *p* = 0.019	ns	ns
Black/African American (vs. Asian)	ns	*β* = 0.566, 95% CI [0.018, 1.114], *p* = 0.043	ns
Caucasian/White (vs. Asian)	ns	*β* = 0.547, 95% CI [0.137, 0.956], *p* = 0.009	ns
Middle Eastern/North African (vs. Asian)	ns	*β* = 0.852, 95% CI [0.068, 1.635], *p* = 0.033	ns
Other demographics	ns	ns	ns
Model fit	*R*^2^ = 0.332	*R*^2^ = 0.307	*R*^2^ = 0.414

Significant demographic covariates in regression models are presented, with standardized *β* coefficients and 95% confidence intervals. All models controlled for age, ethnicity, marital status, occupation, education, and duration of PMOS symptoms. Reference categories: Age 18–24, Ethnicity = Asian. ns, non-significant (*p* > 0.05).

**Table 7:** Collinearity statistics for regression models adjusted for demographics.

Regression model	VIF and tolerance range	Multicollinearity status
PSS: CTQ-SF + demographics	VIF 1.05–1.22	No evidence of problematic multicollinearity
	Tolerance 0.819–0.950	
PCOSQ: CTQ-SF + demographics	VIF 1.05–1.22	No evidence of problematic multicollinearity
	Tolerance 0.819–0.950	
PCOSQ: PSS + demographics	VIF 1.05–1.23	No evidence of problematic multicollinearity
	Tolerance 0.813–0.950	

PSS, Perceived Stress Scale; PCOSQ, Polycystic Ovary Syndrome Health-Related Quality of Life Questionnaire; CTQ-SF, Childhood Trauma Questionnaire–Short Form; VIF, variance inflation factor.

### 3.4 Indirect statistical association analysis

Considering the emergent associations among PMOS-related quality of life, perceived stress, and childhood trauma, we then tested whether perceived stress statistically accounted for part of the association between childhood trauma and PMOS-related quality of life, with results provided in Table 8. The indirect statistical association linking childhood trauma (CTQ-SF), perceived stress (PSS), and PMOS-related quality of life (PCOSQ) was significant using both percentile (Estimate = −0.302, SE = 0.065, 95% CI [−0.447, −0.180], *β* = −0.177, *z* = −4.67, *p* < 0.001) and bias-corrected approaches (Estimate = −0.302, SE = 0.065, 95% CI [−0.456, −0.186], *β* = −0.177, *z* = −4.67, *p* < 0.001). Both bootstrap confidence intervals excluded zero, supporting the presence of a statistically significant indirect association. Higher childhood trauma was associated with poorer quality of life, with perceived stress statistically accounting for part of this association. At the component level, CTQ-SF was positively associated with PSS (*β* = 0.395, *p* < 0.001), indicating that greater trauma exposure was associated with higher perceived stress. PSS was negatively associated with PCOSQ (*β* = −0.449, *p* < 0.001), indicating that higher stress was associated with poorer PMOS-related quality of life. The direct association between CTQ-SF and PCOSQ remained significant after PSS was included in the model (Estimate = −0.337, SE = 0.107, 95% percentile CI [−0.540, −0.125], 95% bias-corrected CI [−0.550, −0.140], *β* = −0.198, *p* = 0.002), indicating that a direct association remained after accounting for perceived stress. Thus, perceived stress statistically accounted for part, but not all, of the association between childhood trauma and PMOS-related quality of life. The total association between CTQ-SF and PCOSQ was also significant (Estimate = −0.639, SE = 0.110, 95% percentile CI [−0.856, −0.426], 95% bias-corrected CI [−0.865, −0.431], *β* = −0.375, *p* < 0.001), further indicating an overall negative association between childhood trauma and PMOS-related quality of life. An additional covariate-adjusted analysis accounting for age, ethnicity, marital status, occupation, educational level, and duration of PMOS symptoms yielded a materially similar indirect statistical association. The adjusted estimate was −0.291 (SE = 0.065, *β* = −0.171, *z* = −4.50, *p* < 0.001), with the 95% bootstrap confidence interval excluding zero using both the percentile [−0.469, −0.154] and bias-corrected [−0.462, −0.149] methods. Thus, the indirect statistical association remained significant after adjustment for the measured demographic and illness-related covariates.

**Table 8:** Unadjusted indirect statistical association between childhood trauma (CTQ-SF) and PMOS-related quality of life (PCOSQ) through perceived stress (PSS).

Association component	Estimate	SE	*z*	*βₛ*	95% CI percentile	95% CI BC	*p*
Indirect association (*a* × *b*)	−0.302	0.065	−4.67	−0.177	[−0.447, −0.180]	[−0.456, −0.186]	<0.001
Direct association (*c*′)	−0.337	0.107	−3.15	−0.198	[−0.540, −0.125]	[−0.550, −0.140]	0.002
Total association (*c*)	−0.639	0.110	−5.80	−0.375	[−0.856, −0.426]	[−0.865, −0.431]	<0.001
Path a (CTQ-SF → PSS)	0.158	0.026	6.17	0.395	[0.107, 0.211]	[0.107, 0.211]	<0.001
Path b (PSS → PCOSQ)	−1.907	0.267	−7.13	−0.449	[−2.392, −1.439]	[−2.371, −1.420]	<0.001

Unstandardized regression coefficients (Estimates), SE, *z*-values, and *p*-values are shown alongside standardized *βₛ* to indicate relative effect sizes. Path a: CTQ-SF → PSS; Path b: PSS → PCOSQ. Direct, indirect, and total association components were estimated with percentile and bias-corrected (BC) 95% confidence intervals based on 5,000 bootstrap resamples.

Statistical path diagrams using unstandardized and standardized coefficients, respectively, further provide a coherent overview of the analytic results. Figure 2A shows that childhood trauma (CTQ-SF) was positively associated with perceived stress (PSS; *a* = 0.16), which in turn was strongly and negatively associated with PMOS-related quality of life (PCOSQ; *b* = −1.91), while the direct association between CTQ-SF and PCOSQ remained negative (*c*′ = −0.34). Figure 2B facilitates comparison of the standardized association estimates: CTQ-SF showed a moderate positive association with PSS (*β* = 0.40), PSS showed a moderate-to-strong negative association with PCOSQ (*β* = −0.45), and the direct association between CTQ-SF and PCOSQ remained significant but smaller in magnitude (*β* = −0.20).

**Figure 2:**
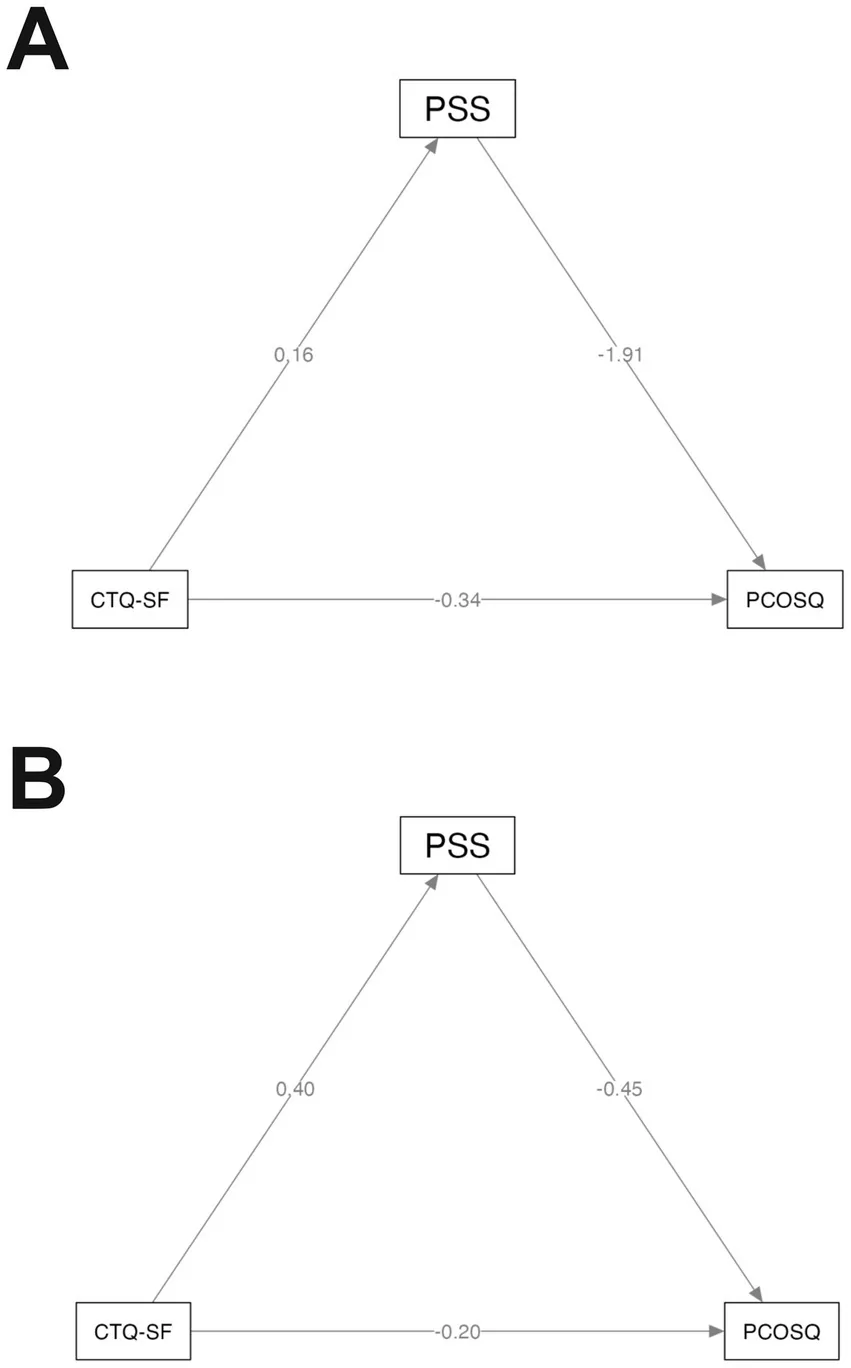
Perceived stress statistically accounts for part of the association between childhood trauma and PMOS-related quality of life. **(A)** displays unstandardized regression coefficients, and **(B)** displays standardized coefficients (*β*), for the unadjusted indirect-association model including childhood trauma (CTQ-SF) as the independent variable, perceived stress (PSS) as the intermediate variable in the statistical model, and PMOS-related quality of life (PCOSQ) as the outcome (*N* = 206). Numbers adjacent to arrows represent path coefficients. Indirect association estimates were obtained using nonparametric bootstrapping with 5,000 resamples; both percentile and bias-corrected 95% confidence intervals are reported. The indirect statistical association was considered statistically significant when the indirect statistical association (*a* × *b*) confidence interval did not include zero (CTQ-SF, Childhood Trauma Questionnaire-Short Form; PSS, Perceived Stress Scale; PCOSQ, Polycystic Ovary Syndrome Quality of Life Questionnaire).

Finally, the estimated indirect, direct, and total association components of the statistical model with corresponding confidence intervals is illustrated in Figure 3. The indirect statistical association (*a* × *b*) was clearly separated from zero. The direct association (*c*′) also remained significant, though smaller in magnitude than the total association (*c* + *a* × *b*). The total association was the largest in magnitude, reflecting the combined magnitude of the direct and indirect statistical associations.

**Figure 3:**
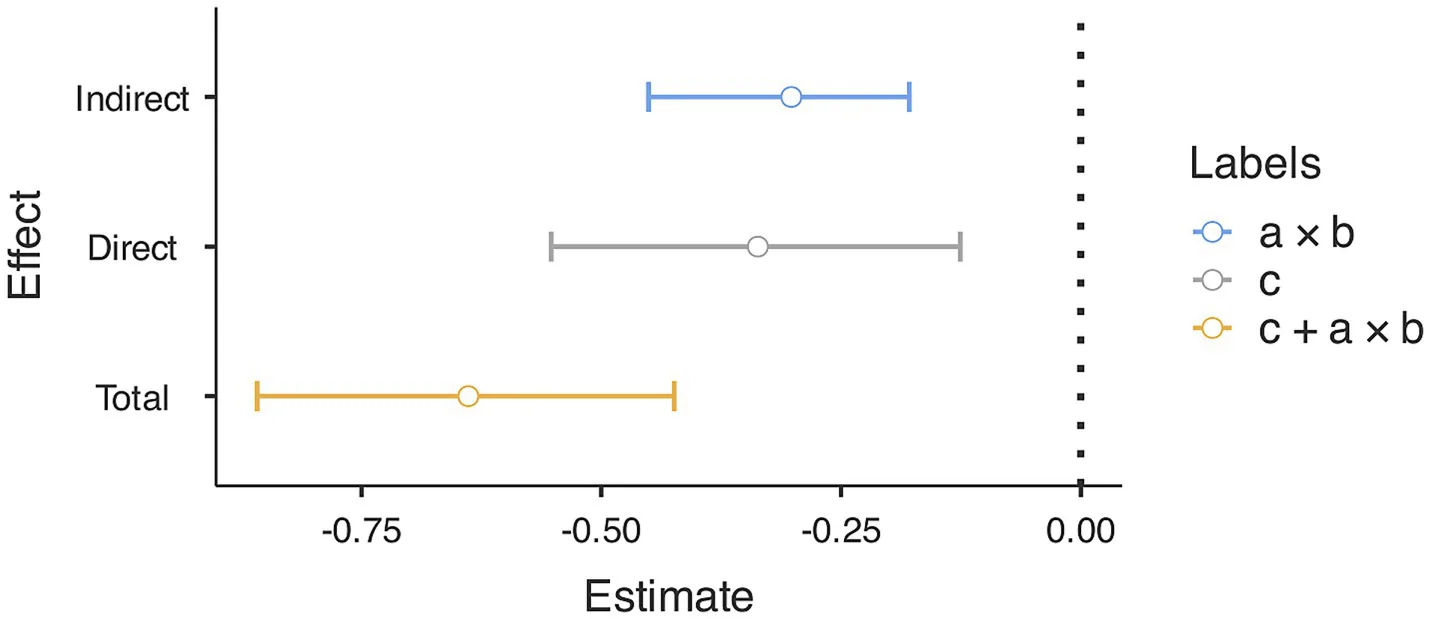
The negative association between childhood trauma and PCOS-related quality of life includes a significant indirect statistical association through perceived stress. Points represent unstandardized estimates of total, direct, and indirect association components derived from the unadjusted indirect-association model (*N* = 206), with horizontal error bars indicating 95% bootstrap confidence intervals. The vertical reference line at zero denotes no association; association estimates are considered statistically significant when confidence intervals do not cross zero. The indirect statistical association was evaluated using nonparametric bootstrapping with 5,000 resamples, and significance was evaluated for the indirect statistical association (*a* × *b*) when its confidence interval excluded zero. (CTQ-SF, Childhood Trauma Questionnaire-Short Form; PSS, Perceived Stress Scale; PCOSQ, Polycystic Ovary Syndrome Quality of Life Questionnaire).

### 3.5 Sensitivity analysis

Sensitivity analyses conducted after excluding cases flagged during the Minimization/Denial re-screening yielded materially similar findings. Childhood trauma remained positively associated with perceived stress (*ρ* = 0.414, *p* < 0.001) and negatively associated with PMOS-related quality of life (*ρ* = −0.399, *p* < 0.001), while perceived stress remained negatively associated with PMOS-related quality of life (*ρ* = −0.507, *p* < 0.001). In the adjusted regression models, childhood trauma remained associated with perceived stress (*β* = 0.359, 95% CI [0.209, 0.508], *p* < 0.001) and PMOS-related quality of life (*β* = −0.382, 95% CI [−0.531, −0.232], *p* < 0.001), while perceived stress remained associated with PMOS-related quality of life (*β* = −0.473, 95% CI [−0.619, −0.328], *p* < 0.001). The indirect statistical association also remained statistically significant and similar in magnitude (Estimate = −0.285, 95% CI [−0.440, −0.159], *p* < 0.001).

## 4 Discussion

This study found that childhood trauma was associated with poorer PMOS-related quality of life and higher perceived stress in women with PMOS, with perceived stress statistically accounting for part of the association between childhood trauma and poorer PMOS-related quality of life.

The association found between greater childhood trauma and higher perceived stress is largely compatible with prior research pointing to trauma-related experiences being linked to enduring differences in responses to subsequent stress. A systematic review of eye-tracking studies in PTSD populations identified scant evidence of enhanced threat detection or generalized hypervigilance, but more consistent evidence was found for sustained attention to threatening information (Lazarov et al., 2019). Convergent physiological evidence from trauma-exposed youth reveals an association between earlier trauma exposure and slower cortisol recovery following acute stress, while associations with diurnal cortisol regulation varied by sex (Kuhlman et al., 2015). These findings are consistent with the broader framework of stress sensitization, whereby lasting alterations in responsivity to later challenges may represent consequences of intense prior stress (Stam et al., 2000). This interpretation should nevertheless remain cautious: a systematic review of PTSD studies produced mixed evidence concerning HPA-axis dysregulation as indexed by diurnal cortisol (Speer et al., 2019). The present study expands the scope of this literature to women with PMOS by signaling the association between greater childhood trauma and higher perceived stress, which was itself associated with poorer PMOS-related quality of life. Given that attentional processing and physiological stress responses were not measured, these prior findings provide possible explanatory context rather than evidence that those mechanisms occurred in the present sample.

At the trauma-subtype level, secondary exploratory analyses showed the largest observed correlations for emotional abuse and emotional neglect with both perceived stress and PMOS-related quality of life. No statistically significant association was detected between sexual abuse and either outcome; however, this null finding should be interpreted cautiously because trauma-subtype prevalence and subtype-specific statistical power were not characterized in the present study. Accordingly, the absence of statistical significance should not be interpreted as evidence that sexual abuse is unrelated to these outcomes. This exploratory pattern can be situated within broader developmental evidence linking childhood maltreatment with difficulties in emotion recognition and regulation, although these processes were not assessed in the present study (Smith and Pollak, 2020). In PMOS-centered research, Simon et al. (2020) observed that anxious and avoidant attachment were associated with psychological distress and that coping strategies partially mediated several attachment-distress associations. Building on these prior insights, the present exploratory findings indicate that greater childhood maltreatment was associated with current perceived stress and PMOS-related quality of life, with the largest observed subtype correlations found for emotional abuse and emotional neglect. Therefore, emotion-regulation, attachment, and coping processes may provide plausible contexts for the interpretation of these associations, but their roles remain to be tested directly.

Prior PMOS-centered research has documented considerable psychological burden and compromised quality of life but has largely focused on the condition’s current clinical and psychological manifestations. Benson et al. (2009), for instance, identified substantially poorer physical and psychological quality of life and greater psychological distress in women with PMOS than in BMI-matched controls, coupled with altered physiological responses to psychosocial stress. Similarly, reviews characterize PMOS as a heterogeneous multisystem condition with relevant psychological sequelae (Joham et al., 2022) and emphasize the nexus between PMOS symptoms and chronic stress with reduced quality of life (Pasquali and Gambineri, 2012). This literature is further extended by the present findings, which highlight that childhood trauma is also associated with poorer PMOS-related quality of life, with a significant direct association retained once perceived stress was included in the statistical model. This implies that perceived stress does not statistically account for the full trauma–quality-of-life association. Therefore, other processes that might account for the remaining association require targeted investigation in future research.

Recent PMOS-specific research yields an especially pertinent comparison for the partial indirect association identified in the present study. Schneider and Chin (2025) found an association between childhood trauma and poorer PMOS-related quality of life, and observed that disengagement coping, but not engagement coping, statistically accounted for a small part of this association; the significance of the direct association between trauma and quality of life was retained. Likewise, the present study identified perceived stress statistically accounting for part of the association between childhood trauma and PMOS-related quality of life. This collective evidence suggests that multiple psychological processes may be involved in the trauma–quality-of-life association in PMOS, with disengagement coping and perceived stress representing potentially distinct, but possibly overlapping, contributors. However, because of the cross-sectional nature of both study designs, these indirect associations cannot establish temporal or causal mechanisms. Longitudinal research could evaluate whether perceived stress and coping, within the same model, make independent or overlapping contributions to PMOS-related quality of life.

Although biological stress-regulatory processes may pertain to the association observed in this study, they were not assessed here. Existing literature provides some foundation for this prospect: childhood trauma has been associated with altered cortisol recovery and diurnal HPA-axis regulation in trauma-exposed youth (Kuhlman et al., 2015), while women with PMOS have shown altered neuroendocrine and cardiovascular responses to acute psychosocial stress (Benson et al., 2009). Nevertheless, the implications for these biological pathways to PMOS-related quality of life have yet to be fully established. A PMOS case–control study found poorer quality of life and higher perceived stress in women with PMOS despite no significant group differences in salivary cortisol or metanephrines following adjustment for age and BMI (Marschalek et al., 2023). Analogously, the present self-report data cannot determine whether biological dysregulation contributes to the association between childhood trauma and PMOS-related quality of life. Future longitudinal, multimethod studies should combine psychological assessments and trauma with biological measures of stress regulation to test whether, and via which biological processes, trauma exposure contributes to PMOS-related quality of life.

The observed indirect statistical association suggests that perceived stress and trauma history may warrant further investigation within research on the psychological assessment and management of PMOS. However, the present cross-sectional findings do not establish that trauma screening or trauma-focused interventions improve PMOS-related outcomes. Prospective and intervention studies are needed to determine whether assessment of perceived stress and trauma history can identify women who may benefit from additional psychological support and whether trauma-informed psychological interventions can reduce perceived stress or improve PMOS-related quality of life. Any future implementation of trauma inquiry in routine PMOS care should involve appropriate consent, clinician training, safeguarding procedures, and access to psychological support.

## 5 Limitations and future directions

Several limitations warrant further examination. The predominantly young, educated, and White sample limits generalizability to more diverse populations, and the cross-sectional design prevents causal inference among trauma, stress, and quality of life in PMOS. Although eligibility demanded participants to report a prior PMOS diagnosis by a healthcare professional, PMOS status was not independently verified by the research team via the retrieval of medical records. Furthermore, in the context of online recruitment, this concern is further pertinent, as enrolment occurred remotely rather than through a clinical setting that could have demonstrated the diagnostic status. Consequently, some degree of diagnostic misclassification cannot be unequivocally excluded and must be considered when interpreting the findings. The exclusion of participants with severe mental health conditions or major physiological comorbidities may also limit generalizability to the broader PMOS population and reduce variability in perceived stress and quality of life, possibly affecting the magnitude of the observed associations. Because 46 of the 252 respondents were excluded before analysis—35 at the eligibility stage and 11 during post-eligibility data-quality screening—selection bias cannot be excluded if excluded respondents differed systematically from those retained in the analytic sample. The use of retrospective self-report measures (CTQ-SF, PSS) increases the risk of recall bias and minimization. The trauma-subtype analyses were exploratory, and trauma-subtype prevalence and subtype-specific statistical power were not specifically characterized; null subtype findings, particularly for sexual abuse, should therefore be interpreted carefully. Additionally, the global PSS does not distinguish between stress domains, such as interpersonal versus health-related stress, which may show different associations with PMOS-related quality of life. Residual clinical confounding remains an important limitation. Although participants with severe diagnosed mental-health conditions and major physiological comorbidities were excluded, potentially relevant variables including BMI, PMOS phenotype and symptom severity, medication, or treatment status, subclinical depression and anxiety symptoms, health behaviors, current life stressors, adverse events, and other medical comorbidities were not comprehensively measured and therefore could not be included in the adjusted analyses. These factors could partially account for the observed associations. Moreover, the absence of physiological biomarkers limits the ability to directly test psychobiological interaction hypotheses.

Future research should prioritize ethnically diverse samples and utilize longitudinal, clinically verified cohorts alongside multi-method mechanistic designs. These designs may include ecological momentary assessment of stress and affect, laboratory-based stress reactivity paradigms, and biomarkers such as cortisol and inflammatory indices. Evaluating protective factors, such as adaptive coping, resilience, and social support, could further clarify potential buffers against trauma-related vulnerability in PMOS. These approaches may help distinguish psychological and biological pathways and their interactions. Randomized intervention trials that assess trauma-informed psychological interventions targeting emotion regulation, maladaptive schemas, and social support in women with PMOS could determine whether reductions in perceived stress mediate improvements in quality of life. Given the exploratory subtype findings, future studies with larger samples, explicit characterization of trauma-subtype prevalence, and adequate subtype-specific statistical power are needed to determine whether different forms of early adversity show distinct associations with perceived stress and PMOS-related quality of life.

## 6 Conclusion

The present study found that poorer PMOS-related quality of life was associated with higher perceived stress and greater childhood trauma, with perceived stress statistically accounting for part of the association between childhood trauma and PMOS-related quality of life. These outcomes underscore the relevance of current perceived stress and trauma history when evaluating psychological well-being and quality of life in women with PMOS. Because of the cross-sectional and self-report design, the findings should be interpreted as associations rather than evidence of causal pathways.

## Data Availability

The datasets presented in this article are not readily available because they are not publicly available as they contain sensitive childhood-trauma and health-related information, and participant-level data are subject to the confidentiality conditions of the informed consent and approved ethics protocol. Requests for aggregated data supporting the findings should be directed to the corresponding author.
